# What Simon “knows” about cultural differences: The influence of cultural orientation and traffic directionality on spatial compatibility effects

**DOI:** 10.3758/s13421-022-01360-9

**Published:** 2022-09-30

**Authors:** Pamela Baess, Ullrich K. H. Ecker, Steve M. J. Janssen, Zheng Jin, Christina Bermeitinger

**Affiliations:** 1 Institute of Psychology, University of Hildesheim, Universitätsplatz 1, 31141 Hildesheim, Germany; 2 International Joint Laboratory of Behavior and Cognitive Science, Zhengzhou Normal University, Zhengzhou, China; 3 Department of Psychology, University of Western Australia, Perth, WA Australia; 4 School of Psychology, University of Nottingham Malaysia, Semenyih, Malaysia; 5 Zhengzhou Normal University, Zhengzhou, China

**Keywords:** Culture, Simon effect, Spatial cognition, Spatial reference frames, Cultural orientation, Traffic directionality, Spatial compatibility

## Abstract

Previous research has suggested that culture influences perception and attention. These studies have typically involved comparisons of Westerners with East Asians, motivated by assumed differences in the cultures’ self-concept or position on the individualism-collectivism spectrum. However, other potentially important sources of cultural variance have been neglected, such as differences in traffic directionality shaped by the urban spatial environment (i.e., left-hand vs. right-hand traffic). Thus, existing research may potentially place too much emphasis on self-concepts or the individualism-collectivism dimension in explaining observed cultural differences in cognition. The present study investigated spatial cognition using a Simon task and tested participants from four nations (Australia, China, Germany, and Malaysia) that differ in both cultural orientation (collectivistic vs. individualistic) and traffic directionality (left-hand vs. right-hand traffic). The task used two possible reference frames underlying the Simon effect: a body-centered one based on global stimulus position relative to the screen’s center versus an object-centered one based on local stimulus position relative to a context object. As expected, all groups showed a reliable Simon effect for both spatial reference frames. However, the global Simon effect was larger in participants from countries with left-hand traffic. In contrast, the local Simon effect was modulated by differences in cultural orientation, with larger effects in participants from collectivistic cultures. This pattern suggests that both sources of cultural variation, viz. cultural orientation and traffic directionality, contribute to differences in spatial cognition in distinct ways.

**Supplementary Information:**

The online version contains supplementary material available at 10.3758/s13421-022-01360-9.

## Introduction

This study is about how individuals from different cultures code space. As a general note, most studies on cultural differences have compared Western and East Asian cultures, focusing on well-known differences in self-concept between independent and interdependent cultures (e.g., Markus & Kitayama, 1991) or differences in cultural orientation (individualism vs. collectivism; e.g., Triandis, 1989). The influence of differences in the spatial environment, however, has largely been ignored in most cross-cultural comparisons (also see Schultz et al., 2012). To illustrate, when thinking about cultural differences in spatial cognition, one could consider the experience of a tourist from mainland Europe or the USA having to adjust to operating a right-hand vehicle in left-hand traffic (e.g., in Australia or Malaysia), or vice versa. This is a challenging task and has been identified as a substantial contributor to traffic accidents (Thompson & Sabik, 2018). As this example illustrates, aspects of the spatial environment vary across countries, as shown by the differences in urban spatial environment for left- and right-hand traffic.

Traffic directionality (i.e., which side of the road is used for driving) is thus one prominent example of a spatial-environment factor that is shaped by cultural background and can influence cognition (Linkov & Zámecník, 2020). Shinohara and Nishizaki (2018) investigated Japanese drivers’ eye movements during simulated driving on Japanese roads (with left-hand traffic) versus foreign roads (with right-hand traffic) and found longer fixation durations in the former condition. The authors suggested that these differences reflected an increased cognitive workload when driving in an unfamiliar driving environment; however, the study could not isolate the specific role of traffic directionality due to the confound with general familiarity. There have also been suggestions that traffic directionality influences travel behavior, as crashes occur more frequently on one side of the vehicle compared to the other (e.g., the left side in right-hand traffic environments; Friedrich et al., 2017). This suggestion makes sense because learned knowledge of spatial urban environment regularities is essential for spatial behavior. To illustrate, parameters of cognitive maps – representations of knowledge regarding travel routes, destinations, distances, etc., that are learned through wayfinding and travel experiences (Golledge & Gärling, 2004) – differ not only on an individual level but also between cultural groups (Kitchin & Blades, 2002).

The present study examined cross-cultural spatial differences alongside differences in cultural orientation to investigate cross-cultural differences in spatial cognition. Differences in spatial factors, such as navigation, spatial knowledge, travel experience, and transport infrastructure, are to some extent underrepresented in cross-cultural research. The focus of the present work was on differences in traffic directionality, as its influence is present from a very young age and, most importantly, as it can be differentiated clearly into left-hand versus right-hand traffic based on a country’s traffic infrastructure. The study’s aim was to concurrently evaluate the impact of both traffic directionality (left-hand traffic vs. right-hand traffic) and cultural orientation (individualism vs. collectivism) in a cross-cultural study of spatial cognition using the Simon task. In the following sections, we briefly summarize cultural differences in spatial cognition before introducing the Simon task used in the present study.

### Cultural differences in perception and cognitive style

The main distinctions drawn in cross-cultural research relate to the different ways in which people conceptualize their selves (Heine & Ruby, 2010) and the emphasis that is placed in a person’s culture on the value of the individual versus the group (i.e., individualism vs. collectivism; Triandis, 1989). An independent self-concept implies that both the self and others are defined by a unique set of characteristics, attitudes, and personality traits, which are seen as stable across situations; this self-concept is prevalent in individualistic cultures that value personal freedom (e.g., North America, Europe, Australia). An interdependent self-concept, on the other hand, implies that the self and others are defined by inter-personal relations, relational roles, and group memberships; behaviors are primarily evaluated with regard to their impacts on others. This self-concept is more prevalent in collectivistic nations in East Asia, where personal preferences are less central than the well-being of a larger group. These distinctions in self-concept and cultural orientation have frequently been used to explain identified cultural differences in psychological processes (for review, see Oyserman et al., 2002). Here, we focus on cultural orientation (individualistic vs. collectivistic), acknowledging that it is not independent of differences in self-concept.

Culture influences various aspects of cognition, including perception and cognitive style (Nisbett & Miyamoto, 2005), attention (Masuda & Nisbett, 2001), and memory (Gutchess & Indeck, 2009). For example, when viewing visual scenes, East Asians tend to exhibit more holistic perception, focusing on relations and contextual elements, whereas Westerners tend to focus on individual objects and their details (for overview, see Masuda & Nisbett, 2001; Nisbett & Masuda, 2003). When changes in a visual scene occur, Westerners tend to detect more changes to salient, focal objects, whereas East Asians are more likely to detect changes in the background or in the relations between objects (Masuda & Nisbett, 2006). Differences in self-concept have also been linked to changes in perceptual biases in spatial illusions (Krishna et al., 2008).

Differences in attentional processes were explored by Boduroglu et al. (2009) using a change detection task. Compared to Westerners, East Asians showed superior change detection when wider visual displays were used, but inferior performance with narrower visual displays, suggesting a cultural influence on attention allocation (but see Boduroglu & Shah, 2017, and Hakim et al., 2017, for contrasting findings). However, these differences in the attentional focus may not generalize to differences in other tasks; for example, Lawrence et al. (2020) found no differences in the distribution of covert spatial attention in Posner’s cueing paradigm in a diverse sample of participants recruited in China and Australia.

Differences between individualistic and collectivistic countries have been observed for various factors influencing traffic behavior and driving behavior (e.g., Lee et al., 2020; Linkov & Zámecník, 2020; Nordfjaern et al., 2014; Ozkan et al., 2006; Pele et al., 2017; Shinohara & Nishizaki, 2018). This line of research explains cultural differences between individualistic and collectivistic countries with the described differences in cognitive style. Consequently, a higher focus on detail may be beneficial in situations where detection of specific details in the environment is important, such as the vehicle’s brake lights or indicators lighting up, explaining why Westerners outperform Easterners in these aspects of traffic behavior. By contrast, Easterners might show performance advantages in situations that require tracking of many objects at once, as is the case in dense traffic. Linkov and Zámecník (2020) promoted the idea that people from holistic cultures have problems with driving in analytical countries and vice versa, which they linked to drivers’ differential expectations and cognitive styles.

### Cultural differences in spatial cognition

How we use and represent space and spatial information has been investigated in different fields of research, including anthropology, linguistics (e.g., Levinson, 2003), psychology, as well as geography and traffic planning (e.g., Kitchin & Blades, 2002). One fundamental aspect of representing and communicating spatial relations, including person-to-object and object-to-object relationships, is the reference frame used, which determines the spatial coordinate axes in relation to which an object or a person is located. Different spatial reference frames are used to organize the environment in such a way that the exact positions or coordinates of objects in their visual field can be described (for review, see Filimon, 2015; Humphreys et al., 2013; Klatzky, 1998; Proulx et al., 2016).

One school of thought differentiates between two different spatial coordinate systems: a self/body-centered (egocentric) reference frame focuses on the viewer’s perspective, and a person may describe an object’s position as “on my left” or “in front of me.” This way of organizing the spatial environment emphasizes the spatial features of an object in reference to the viewer. In contrast, when the position of a certain object is given relative to another object – for example, an object’s position may be described as “in front of a house” – more local features of the object’s position are emphasized. This is an object-centered reference frame and less dependent on viewpoint; it is sometimes referred to as allocentric. This classification is commonly used in navigation, wayfinding, perspective taking, and psychology.

However, linguists discuss three different coordinate systems (Levinson, 1996; Majid et al., 2004): relative, intrinsic, and absolute. A relative coordinate system is based on the speaker’s view (corresponding to the self-centered reference frame). An intrinsic coordinate system uses the location of other objects as a reference for direction (roughly corresponding to the object-centered reference frame). The absolute coordinate system refers to absolute cardinal directions (e.g., “North of the lake”). Majid et al. (2004) proposed that the latter two can be considered distinct types of allocentric reference frames. Importantly, previous research has highlighted cultural differences in the linguistic coding of space and in the usage of these reference frames in particular (Levinson, 2003). Some cultures may use all three linguistic references frames (albeit to varying extent), whereas others focus only on one or two reference frames when describing spatial positions. Thus, language and other cultural factors may modulate choice of the preferred reference frame (Levinson et al., 2002; Pederson et al., 1998).

Another line of research has investigated effects of spatial stimulus-response (SR) compatibilities, linking stimulus perception (S) with the appropriate response (R). Some SR combinations can be processed more easily (i.e., when features of the stimulus and the response are compatible) or less easily (i.e., when features of the stimulus and response are incompatible), because of the particular set of stimuli and responses used or because of the arbitrary mapping between them (Kornblum et al., 1990). To illustrate, cultural differences have been identified with the global/local task (Navon, 1977). This task investigates compatibilities at the level of stimulus identification, as global and local stimulus features overlap or not. Specifically, a large stimulus composed of small stimuli (e.g., a large letter composed of small letters) is presented and participants make an identity judgment regarding either the large letter or the small letters. Typically, a global precedence effect is observed, with faster judgment of global (i.e., the large stimulus) than local stimulus identities (i.e., the small stimuli), as well as a global-local interference effect, with slower responses to incompatible trials (e.g., a large ‘T’ made up of small ‘S’s) than to compatible trials (e.g., a large ‘T’ made up of small ‘T’s). A stronger global precedence effect (McKone et al., 2010) and stronger interference (Wong et al., 2021) has been found for East Asian compared to Western participants.

### Cultural differences in the Simon task

Despite the evidence for cultural influences on SR compatibilities at the early stimulus identification stage, it remains unclear whether cultural differences generalize to cognitive control processes underlying goal-directed behavior, including response selection and response inhibition processes. One prominent paradigm investigating response selection at the interface of perception and action is the Simon task (Hommel, 2011; Proctor, 2011; Simon, 1990). In the Simon task, participants classify stimuli into two categories based on a task-relevant stimulus feature (e.g., color or shape), while being instructed to ignore task-irrelevant stimulus features including spatial location. An arbitrary stimulus-response mapping is implemented (e.g., left-hand response key for blue stimuli and right-hand response key for yellow stimuli). SR compatibility is based on the feature codes of the stimulus location (e.g., stimulus displayed on left or right side of the screen) and the feature codes of the response location (e.g., left or right key presses). Typically, performance is better if task-irrelevant spatial location is compatible with the response mapping (e.g., blue stimuli presented on the left of the screen) rather than incompatible (e.g., blue stimuli presented on the right); this is called the Simon effect.

The Simon effect can be explained by a conflict between dual processing routes: a controlled route based on instruction (e.g., “blue is left”) and an automatic route driven by the overlap of spatial stimulus and response features (e.g., a stimulus presented on the right-hand side facilitating a response with the right hand; De Jong et al., 1994). According to the theory of event coding (TEC; Hommel, 2019; Hommel et al., 2001), different stimulus and response feature codes are stored together as event files, consisting of integrated networks of sensorimotor feature codes. The event files are weighted intentionally so that those features that are more salient with regard to the current intention receive a stronger weight (Memelink & Hommel, 2013). One might argue that Simon effects indicate the relative salience of the horizontal (left/right) spatial position, linked to a self-centered (body-midline) reference frame. However, Simon effects can also be obtained along the vertical axis, and even with different spatial codes defining the reference frame (for review, see Rubichi et al., 2006). The Simon effect can also be used as an indicator of different reference frames being applied that are linked to differences in the processing of global and local stimulus features (Baess & Bermeitinger, 2022; Baess et al., 2022; Lamberts et al., 1992; Nicoletti & Umilta, 1984; Roswarski & Proctor, 1996; Rubichi et al., 2006; Wang et al., 2016). Simon effects (and spatial compatibility effects in general) are universal, as Simon effects have been found in different samples in Western and Asian countries (for an overview, see Proctor & Vu, 2010), which points to the general nature of the processes underlying the emergence and resolution of response conflict in spatial cognition. However, few studies have explored cultural influences on the Simon effect and on processes of response selection more generally.

Some initial evidence has shown performance differences on the Simon task between religious and non-religious participants, and between monolinguals and bilinguals. For example, Hommel et al. (2011) found that relative to a non-religious control group, Calvinists (who tend to emphasize individualism) showed smaller Simon effects, and Catholics (who tend to emphasize collectivistic ideas) showed larger Simon effects. Samuel et al. (2018) obtained a smaller Simon effect in a group of collectivistic (Korean) participants compared to an individualistic (British) group.

Taken together, there are well-described cultural differences in stimulus-response compatibility task performance covering early stimulus identification and response selection processes. The common procedure in most of these studies is that different countries were selected that varied on the collectivism-individualism spectrum (see also Hofstede, 2001), and comparisons were made between Western and East Asian cultures. Yet, for spatial cognition, and more specifically spatial compatibility, cultural differences in spatial factors might be more relevant. For example, the cross-cultural differences in the Simon task previously reported and attributed to differences in cultural orientation between the UK and Korea (Samuel et al., 2018) may also be caused by cultural differences in traffic directionality (i.e., left-hand vs. right-hand traffic). The problem that two cultures differ in more than just the intended factors is a common one for cross-cultural comparisons, and it seems particularly critical for existing spatial cognition research. The problem could be addressed by incorporating additional factors (e.g., traffic directionality) alongside established factors (e.g., cultural orientation) into cross-cultural study designs to allow for stronger conclusions regarding the underlying cognitive mechanisms and avoid potential circularity problems (see Alotaibi et al., 2017, for a related argument). As stated elsewhere (Wang, 2017), cross-cultural research is not just about documenting observable differences between cultures, but also about exploring differences in (cognitive) mechanisms underlying the observed differences.

Bearing in mind the goal to examine underlying mechanisms, traffic directionality is a potentially relevant factor for spatial compatibility tasks. Traffic directionality can be easily determined, shapes the spatial environment (including traffic infrastructure, interior vehicle layouts, and the urban environment more generally), its influence is similar for all individuals of a country, and it has been identified as a factor that influences spatial cognition and behavior (e.g., travel accidents; Thompson & Sabik, 2018). However, to the best of our knowledge, the impact of traffic directionality on spatial cognition has not been directly compared between different cultures. We reasoned that traffic directionality may influence the Simon effect by modulating its underlying spatial representations.

### The present study

The present study set out to investigate Simon effects in a cross-cultural study including countries that vary in traffic directionality and cultural orientation. We used the same study protocol in four countries that differed in dominant cultural orientation (individualistic [Australia, Germany] vs. collectivistic [China, Malaysia]) and traffic directionality (left-hand traffic [Australia, Malaysia] vs. right-hand traffic [China, Germany]). As all participants were tested locally, they were not aware of the fact that cultural comparisons were the target of the study. Moreover, nonlinguistic stimuli were used to avoid issues with linguistic differences. To account for differences in cognitive style between Western and East Asian cultures (i.e., analytic vs. holistic perceptual processing; for review, see Nisbett & Miyamoto, 2005), we used two display variants, either presenting one single stimulus (fostering analytical processing as no context objects were given) or a group of identical stimuli (enabling holistic processing of task-irrelevant background information provided through the group arrangement). The stimuli were stick-figure manikins, and the task-relevant feature was the color of a ball (blue or yellow) that each manikin was holding in one hand. This followed the procedure used in other studies in our lab (Baess & Bermeitinger, 2022; Baess et al., 2022; Baess et al., 2018), where using these materials concurrently yielded two kinds of Simon effect: one based on the position of the ball relative to the center of the screen (the “global” Simon effect) and one based on the position of the ball relative to the stick-figure manikin (the “local” Simon effect). We propose that the two types of Simon effect can serve as indicators of different spatial reference frames – a self-centered reference frame anchored at the screen’s center (corresponding to the body midline) versus an object-centered reference frame anchored at the stick-figure manikin. Our previous studies showed a dependency between the visual display (single stimulus vs. group of identical stimuli) and the size of the global Simon effect, in that the global Simon effect decreased with a group display, whereas the local Simon effect remained unaffected by display size.

We expected robust Simon effects in all groups, given its universal nature. Moreover, we expected a modulation of the global and local Simon effects based on cultural variations in cognitive control processes that reflect differences in attention allocation to global and local features during response selection. More specifically, we expected three findings. First, based on previous research reporting cultural differences in the processing of visual displays (Nisbett & Miyamoto, 2005), we expected (South-)East Asians to benefit from a group display that provides an enriched visual context and facilitates holistic processing. This would be supported by shorter RTs for the East Asians in the visual display with a group of stimuli. Second, as East Asians tend to focus more on the relationship between objects and the context in which objects are located, we expected larger local Simon effects for East Asians. Third, given Westerners’ preference to process objects independent of context, we expected them to show a larger global Simon effect with a single-stimulus display. In terms of traffic directionality, we anticipated that it would most likely influence the global Simon effect, as both the stimulus and spatial presentation emphasize global features (i.e., the stimulus position on the screen and general parameters of the spatial environment, respectively). Modulations in the size of the global and local Simon effect between Westerners and East Asians would indicate that the relative weight of a reference frame as the source of the Simon effect can change depending on differences linked to cultural orientation or traffic directionality.

## Method

### Participants

In total, 210 participants were recruited for the present study. One participant did not complete all experimental tasks. Two additional participants were excluded from analyses because of extreme reaction time (RT) outliers (involving more than 40% of all trials). The final sample included 207 participants, consisting of 60 Australian participants from the University of Western Australia (19 female, 41 male; 18–32 years of age; mean age = 19.52 years, *SD* = 2.56); 51 Chinese participants from Zhengzhou Normal University (44 female, three male, four participants of undisclosed gender; 18–23 years of age; mean age = 21.22 years, *SD* = 1.35); 46 German participants from the University of Hildesheim (39 female, seven male; 18–40 years of age; mean age = 20.89 years, *SD* = 3.88); and 50 Malaysian participants from the University of Nottingham Malaysia (28 female, 22 male; 17–43 years of age; mean age = 21.68, *SD* = 4.62). Of all participants, 191 were right-handed as assessed with a Handedness Inventory (Oldfield, 1971): Australia: 51 right-handed, four left-handed, five ambidextrous, mean handedness score: 54.33 (± 6.14 SEM); China: 50 right-handed, one ambidextrous; mean handedness score: 57.75 (± 2.63 SEM); Germany: 43 right-handed, three left-handed; mean handedness score: 71.52 (± 6.41 SEM); Malaysia: 47 right-handed, three left-handed, mean handedness score: 64.00 (± 6.07 SEM). Further details regarding the languages spoken and the reading directions of the four countries can be found in the Online Supplementary Material (OSM), Table 1.

Minimum sample size, calculated with G*Power (Faul et al., 2007), was 128 for a within-between interaction in a mixed design with an effect size of *f* = 0.25, α = .05, and 1-β = .80. Therefore, the total sample size achieved allowed for detection of differences in the Simon effects based on the between-subjects factors traffic directionality and cultural orientation. Participants received either partial course credit (Germany; Australia) or a monetary compensation (China: CNY5; Malaysia: MYR15). All participants had normal or corrected-to-normal vision and gave informed consent to the terms of data collection, use, and storage in accordance with the Declaration of Helsinki. The study was approved by each local university’s ethics board.

### Design

The study comprised a 2 (Display Size: 1 vs. 9) × 2 (Ball Position Compatibility: compatible vs. incompatible) × 2 (Screen Position Compatibility: compatible vs. incompatible) × 2 (Traffic Directionality: left-hand traffic vs. right-hand traffic) × 2 (Cultural Orientation: individualistic vs. collectivistic) design, whereby Display Size, Ball Position, and Screen Position were within-subjects factors and Traffic Directionality and Cultural Orientation were between-subjects factors. Both between-subject factors were fully crossed by using participant groups from four different cultures. Dependent variables were RTs as well as error rates. Simon effects were calculated as the difference between incompatible trials minus compatible trials, separately for Ball Position Compatibility (local Simon effect) and Screen Position Compatibility (global Simon effect).

### Stimuli and experimental conditions

**Fig. 1:**
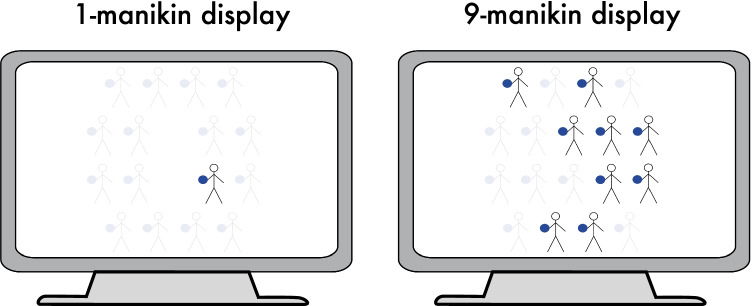
Stimulus setup in both Display Size conditions. **Left:** One stick-figure manikin is presented at one of the 16 spatial positions distributed equally around the screen’s center (1-manikin display). **Right:** A perceptual group of nine identical stick-figure manikins is presented, in a way that a majority of manikins is located on one side of the screen. Note that faint manikins serve only to illustrate the possible positions (see text for further details)

Two-dimensional drawings of stick-figure manikins (see Fig. 1) holding a colored ball (blue: RGB values 0, 0, 255; yellow: 255, 255, 0) in one hand were used in accordance with previous studies (Baess & Bermeitinger, 2022; Baess et al., 2022; Baess et al., 2018). In the stimulus presentation software, the size of the manikin was set to 89 pixels width (2 cm width on a 16-in. laptop screen) and 137 pixels height (3.3 cm height on a 16-in. laptop screen).^1^ The diameter of the colored ball was 0.7 cm. The black lines (RGB 0, 0, 0) were less than 1 mm on the screen.

Eighteen different screen positions were predefined; eight were on the left side of the screen’s vertical midline, eight were on the right side; two positions were exactly on the midline. The exact stimulus positions varied from trial to trial. Display size (one vs. nine manikins) varied between blocks; one-manikin displays randomly used one of the 16 lateralized positions (see left-hand column of Fig. 1), nine-manikin displays randomly used nine of the 18 positions with the constraint that placements were asymmetric, with between four and seven manikins placed on the dominant side and one to four on the opposite side (with none to two placed on the midline). No symmetric sets were used.

The stick-figure manikins themselves simultaneously allowed for spatial coding along two different reference frames – based on the global feature of the screen’s vertical midline (self-centered reference frame; global Simon effect) and the local feature of the ball’s position relative to the manikin’s body midline (object-centered reference frame; local Simon effect). Both reference frames were independently present in any given trial as it was possible to classify each single stimulus or stimulus set in regard to both reference frames. Thus, there were four different conditions differentiating SR compatibility and SR incompatibility for both different reference frames, which is the result of fully crossing the factors Ball Position (compatible, incompatible) and Screen Position (compatible, incompatible).

### Procedure

Data collection took place at the corresponding university campuses in Australia, China, Germany, and Malaysia. Participants were initially provided with an information sheet and provided informed consent; they then received written instructions (in English) on the screen, supplemented by oral instructions in the local language. The experiment was conducted in a sound-shielded testing booth using 15.6-in. Lenovo ThinkPad Laptops (Germany, Malaysia) or a desktop Windows PC with a 21-in. LED screen (Australia), or in a laboratory with multiple working desks, separated by partition walls, using 21-in. LED monitors (China). Distance from the screen was approximately 60–65 cm at each location. The experiment was run using Presentation software (Neurobehavioral Systems, Version 18).

Each trial started with a white fixation cross against a gray background (RGB 132, 132, 132), presented centrally for 500 ms; this was followed by the presentation of the manikin display, which remained on screen for 2,500 ms or until a response was made. After a 1,500-ms blank screen, the next trial started. The participant’s task was to respond to the color of the ball (blue vs. yellow) using their index fingers and the ‘F’ (left) and ‘J’ (right) keys of a standard QWERTY/QWERTZ keyboard. The mapping between stimulus color and left/right response keys was counterbalanced across participants.

The experiment began with a short training phase (16 trials) to familiarize participants with the task; visual accuracy feedback was provided at the end of the training phase. The main experiment consisted of two parts (one for each Display Size condition, with order counterbalanced), each comprising three blocks of 64 trials – eight trials per factorial combination of Screen Position, Ball Position, and Color – for a total of 384 experimental trials. Participants were able to take short, self-paced breaks between blocks. At the end of the experiment, Australian and Chinese participants answered an electronic version of a self-report collectivism-individualism scale (Singelis et al., 1995) (see OSM Table 2).^2^

### Analysis

Reaction time and accuracy were assessed with ANOVAs in SPSS. ANOVAs included Display Size, Ball Position Compatibility, and Screen Position Compatibility as within-subject factors and Traffic Directionality and Cultural Orientation as between-subject factors. In addition, we also analyzed the data with linear mixed-effects models (LMMs) using *lme4* (Bates et al., 2015) in the *R* environment (R Core Team, 2012) to enable a comparison with other statistical analysis procedures. The LMMs used Ball Position Compatibility, Screen Position Compatibility, Display Size, Traffic Directionality, and Cultural Orientation as fixed effects and participant ID as random effects along with random slopes for the effect of Display Size on participants. Hypothesis tests were performed using the *Anova* function of the *car* package (Fox & Weisberg, 2019). Accuracy was assessed using binomial generalized linear mixed-effects models (GLMM). Based on our research interest, we fit only a model including all fixed-effect interactions.

## Results

Analyses were conducted separately on mean RTs and error rates. Only significant effects (*p* < .05) are reported unless the non-significant effects were deemed informative. Bonferroni-corrected *p*-values were used for post hoc tests. To additionally validate our results, we also analyzed *z*-transformed Simon effects in RTs to rule out an influence of overall processing speed.

### Reaction times

Only correct responses with RTs above 100 ms and below 1.5 interquartile ranges above the third quartile of the individual RT distribution (Tukey, 1977) were used for the RT analysis. Averaged across all participants, 93.34% of all trials were included in the RT analysis: no responses were obtained in 0.11% of all trials, 1.87% of all trials were excluded because of erroneous responses, and 4.69% of all trials were identified as response outliers.

**Fig. 2:**
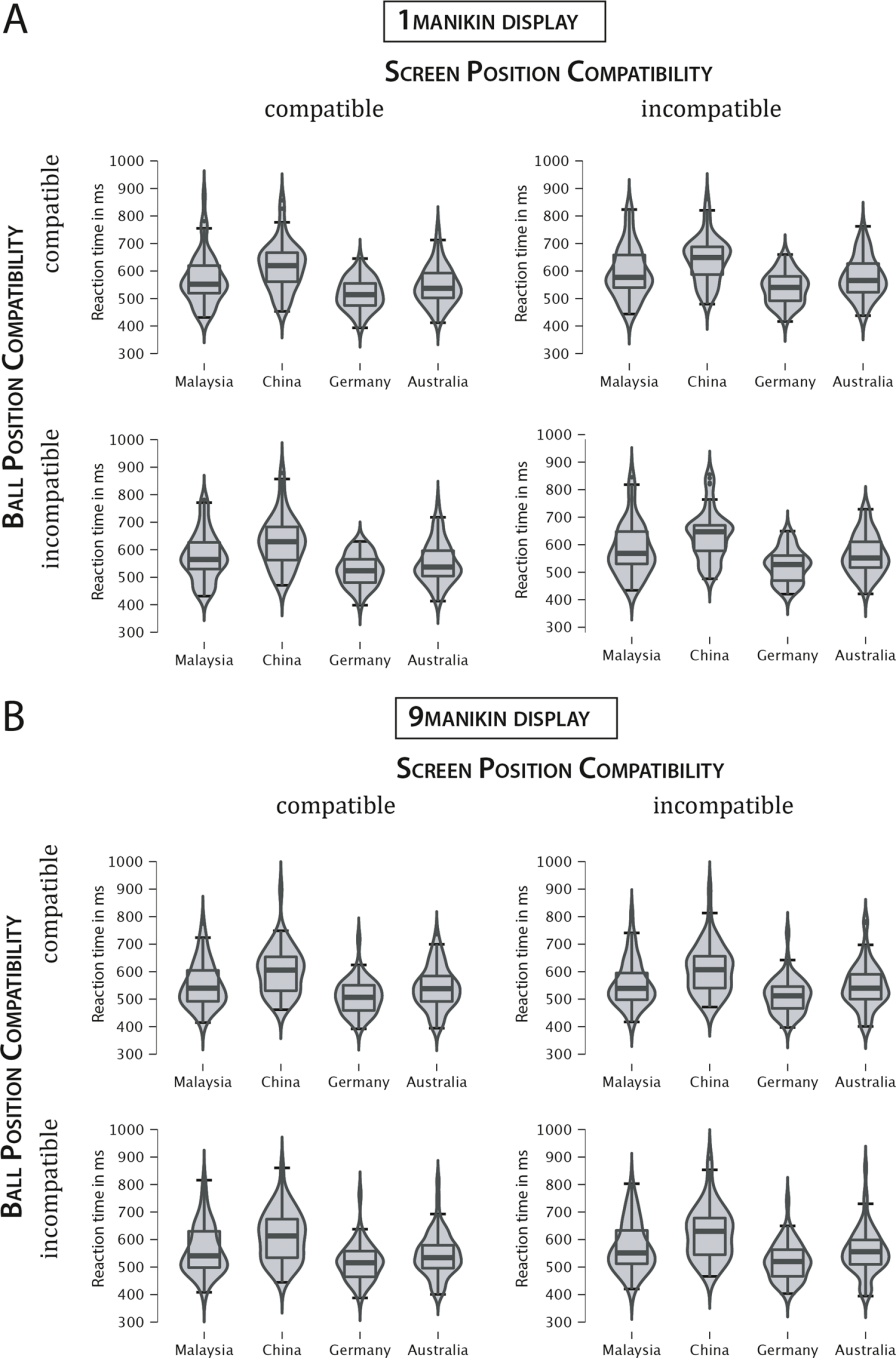
Reaction times are shown as a violin plot with box plots for the four countries as a function Screen Position Compatibility, and Ball Position Compatibility separately for Display Size (1-manikin [top row] vs. 9-manikin display [bottom row]). The countries were analyzed in regard of the traffic directionality (left-hand traffic [Australia, Malaysia] vs. right-hand traffic [China, Germany]) and cultural orientation (collectivism [China, Malaysia] vs. individualism [Australia, Germany]). The box shows the interquartile range with the central bar depicting the median and the whiskers showing 1.5 x the interquartile range from the first and third quartiles, respectively

The overall ANOVA revealed three significant main effects (see Fig. 2): a main effect of Display Size, *F*(1, 203) = 73.89, *p* < .001, ƞ_p_^2^ = .267, indicating faster responses for the nine-manikin display (*M* = 559 ms; *SEM* = 6 ms) than the one-manikin display (*M* = 579 ms; *SEM* = 6 ms); a main effect of Ball Position Compatibility, *F*(1, 203) = 90.21, *p* < .001, ƞ_p_^2^ = .308, which demonstrates a local Simon effect with faster responses for compatible trials (*M* = 565 ms; *SEM* = 6 ms) compared to incompatible trials (*M* = 574 ms; *SEM* = 6 ms); and a main effect of Screen Position Compatibility, *F*(1, 203) = 390.20, *p* < .001, ƞ_p_^2^ = .658, revealing a global Simon effect with faster responses for compatible trials (*M* = 561 ms; *SEM* = 6 ms) than incompatible trials (*M* = 577 ms; *SEM* = 6 ms).^3^

The global Simon effect depended on display size, as revealed by a significant interaction of Screen Position Compatibility and Display Size, *F*(1, 203) = 131.23, *p* < .001, ƞ_p_^2^ = .393. Larger global Simon effects were observed for the one-manikin display (*M* = 25 ms; *SEM* = 1 ms) compared to the nine-manikin display (*M* = 7 ms; *SEM* = 1 ms), *t*(207) = 11.67, *p* < .001. The interaction of Display Size and Ball Position Compatibility, indicating differences in the local Simon effect, was non-significant, *F*(1, 203) = 3.63, *p* = .058, ƞ_p_^2^ = .018. These results are comparable to those observed in other work of our group with a two-choice Simon task (Baess & Bermeitinger, 2022; Baess et al., 2022) and even comparable to our results found in a Go/No-Go version of this task (Baess et al., 2018).

Of particular interest are the effects including the between-subjects factors Cultural Orientation and Traffic Directionality. The main effect of Cultural Orientation was significant, *F*(1, 203) = 29.37, *p* < .001, ƞ_p_^2^ = .126, suggesting that individualistic cultures (Australia, Germany) were associated with faster responses (*M* = 542 ms; *SEM* = 7 ms) compared to collectivistic cultures (China, Malaysia; *M* = 598 ms; *SEM* = 9 ms). The main effect of Traffic Directionality was non-significant, *F* < 1. More interestingly, there was a significant interaction between Traffic Directionality and Cultural Orientation, *F*(1, 203) = 15.03, *p* < .001, ƞ_p_^2^ = .069, which was further decomposed with post hoc *t*-tests: RTs were lowest in participants with right-hand traffic and individualistic culture (Germany; *M* = 523 ms; *SEM* = 9 ms), which differed significantly from the group with right-hand traffic and collectivistic culture (China; *M* = 623 ms; *SEM* = 12 ms), *t*(95) = 6.68, *p* < .001. Significant differences between left-hand traffic and right-hand traffic emerged (in opposite directions) for both individualistic cultures (Australia vs. Germany), *t*(103) = 2.51, *p* = .014, and collectivistic cultures (Malaysia vs. China), *t*(99) = 2.94, *p* = .004.

**Fig. 3:**
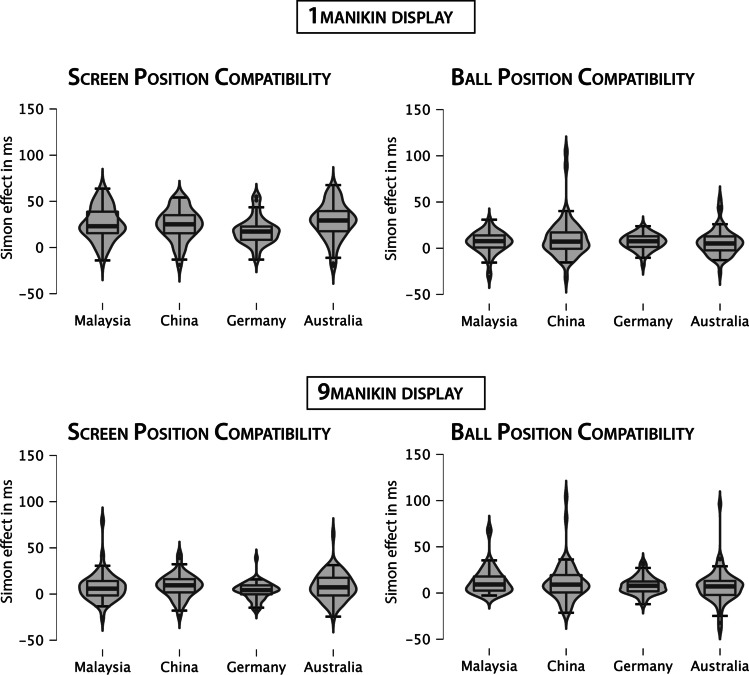
Simon effects by country and Display Size (1-manikin [top row] vs. 9-manikin display [bottom row]); Simon effects indicate the difference between incompatible and compatible trials, based on the global, self-centered reference frame of the screen’s center (global Simon effect, left column) and the local, object-centered reference frame of the manikin (local Simon effect, right column). The box shows the interquartile range with the central bar depicting the median and the whiskers showing 1.5 x the interquartile range from the first and third quartiles, respectively

The global Simon effect was modulated by traffic directionality, as evident from a significant interaction of Screen Position Compatibility and Traffic Directionality,^4^*F*(1, 203) = 5.06, *p* = .026, ƞ_p_^2^ = .024, indicating a larger global Simon effect in participants with left-hand traffic (Australia, Malaysia). This effect was further qualified by a three-way interaction involving Cultural Orientation, *F*(1, 203) = 5.45, *p* = .021, ƞ_p_^2^ = .026. For the local Simon effect, the interaction between Ball Position Compatibility and Cultural Orientation was non-significant, *F*(1, 203) = 3.83, *p* = .052, ƞ_p_^2^ = .018. Follow-up analyses were nevertheless conducted with both global and local Simon effects (see Fig. 3). The global three-way interaction was decomposed by stepwise post hoc grouped *t*-tests. In participants with an individualistic culture (Australia, Germany), larger global Simon effects were observed with left-hand traffic (Australia; *M* = 18 ms; *SEM* = 2 ms) compared to right-hand traffic (Germany; *M* = 11 ms; *SEM* = 1 ms), *t*(104) = 3.23, *p* = .002. In a similar vein, the global Simon effects differed between individualistic (Germany; *M* = 11 ms; *SEM* = 1 ms) and collectivistic (China; *M* = 17 ms; *SEM* = 1 ms) cultures with right-hand traffic, *t*(104) = 3.01, *p* = .003, but not for cultures with left-hand traffic (Australia, Malaysia), *t* < 1. The observed interaction between Ball Position Compatibility and Cultural Orientation indicates that the local Simon Effect was larger for collectivistic cultures (China, Malaysia; *M* = 11 ms; *SEM* = 2 ms) than individualistic cultures (Australia, Germany; *M* = 7 ms; *SEM* = 1 ms), *t*(205) = 2.00, *p* = .046.

To check if the observed cultural differences could be related to overall RT differences between the four groups of participants, exactly the same analysis was conducted on normalized RT data (z-scores with μ = 0 and σ = 1) whereby outliers were identified as being larger than 2.5 scores. The overall ANOVA obtained similar results: a main effect of Display Size, *F*(1, 203) = 89.80, *p* < .001, ƞ_p_^2^ = .307, a main effect of Ball Position Compatibility, *F*(1, 203) = 123.31, *p* < .001, ƞ_p_^2^ = .378, a main effect of Screen Position Compatibility, *F*(1, 203) = 418.55, *p* < .001, ƞ_p_^2^ = .673, and a main effect of Cultural Orientation, *F*(1, 203) = 28.67, *p* < .001, ƞ_p_^2^ = .124. Three interactions were significant, namely those between Display Size and Screen Position Compatibility, *F*(1, 203) = 122.55, *p* < .001, ƞ_p_^2^ = .376; between Screen Position Compatibility and Traffic Directionality, *F*(1, 203) = 10.15, *p* = .002, ƞ_p_^2^ = .048, and between Traffic Directionality and Cultural Orientation, *F*(1, 203) = 14.50, *p* < .001, ƞ_p_^2^ = .067. With the normalized dataset, the ANOVA did not show any interactions between Cultural Orientation and Screen or Ball Position Compatibility. Taken together, the analysis with the normalized dataset implies that overall differences in speed between the four countries cannot explain the culturally shaped modulations of the Simon effects.

The LMM was run on trial-wise RT data using the following model specifications:

- lmer(RT ~ Screen Position Compatibility * Ball Position Compatibility * Display Size * Traffic Directionality * Cultural Orientation + (1 + Display Size | ParticipantID), REML=TRUE).

This model replicated the ANOVA results, with significant fixed effects of Display Size, χ^2^(1) = 28.78, *p* < .001, showing faster responses in the nine-manikin display; Screen Position Compatibility, χ^2^(1) = 352.38, *p* < .001, showing faster responses for Screen Position compatible trials; Ball Position Compatibility, χ^2^(1) = 141.11, *p* < .001, showing faster responses for Ball Position compatible trials, and the fixed effect of Cultural Orientation, χ^2^(1) = 24.85, *p* < .001, showing faster RTs for individual cultures (Australia, Germany). Moreover, in line with the ANOVA results, the interactions between Traffic Directionality and Cultural Orientation, χ^2^(1) = 16.23, *p* < .001; Screen Position Compatibility and Traffic Directionality, χ^2^(1) = 8.92, *p* = .002; Screen Position Compatibility and Display size, χ^2^(1) = 78.91, *p* < .001; and Ball Position Compatibility and Cultural Orientation, χ^2^(1) = 8.13, *p* < .004, were significant. In addition, this model found a three-way interaction between Screen Position Compatibility, Display Size, and Traffic Directionality, χ^2^(1) = 5.10, *p* = .024.

### Error rates

Analysis of error rates excluded trials without a response, given their low frequency. The overall ANOVA produced a similar pattern to the RT analysis, yielding the same three main effects: a main effect of Display Size, *F*(1, 203) = 9.82, *p* = .002, ƞ_p_^2^ = .046, with fewer errors for the nine-manikin display (*M* = 1.50%; *SEM* = 0.17%) than the one-manikin display (*M* = 2.23%; *SEM* = 0.23%); a main effect of Ball Position Compatibility, *F*(1, 203) = 19.99, *p* < .001, ƞ_p_^2^ = .090, indicating a local Simon effect with fewer errors for compatible trials (*M* = 1.33%; *SEM* = 0.12%) than incompatible trials (*M* = 2.40%; *SEM* = 0.27%); and a main effect of Screen Position Compatibility, *F*(1, 203) = 70.02, *p* < .001, ƞ_p_^2^ = .256, indicating a global Simon effect with fewer errors for compatible trials (*M* = 1.32%; *SEM* = 0.16%) than incompatible trials (*M* = 2.41%; *SEM* = 0.20%).^5^

Three two-way interactions were significant: The first one was the interaction between Screen Position Compatibility and Display Size, *F*(1, 203) = 34.69, *p* < .001, ƞ_p_^2^ = .146; this finding mirrored the RT analysis and indicated a larger global Simon effect in the one-manikin display than the nine-manikin display (*M* = 1.72%; *SEM* = 0.20% vs. *M* = 0.48%; *SEM* = 0.13%, respectively), *t*(206) = 6.04, *p* < .001. The second one was an interplay between both global and local Simon effects as shown by the interaction between Ball Position Compatibility and Screen Position Compatibility, *F*(1, 203) = 11.29, *p* < .001, ƞ_p_^2^ = .053. Pairwise *t*-tests were calculated for all possible combinations of Ball Position Compatibility and Screen Position Compatibility. All but one comparison were highly significant, *t*(206) ≥ 2.88, *p* ≤ .004, the exception being the contrast of compatible ball position/incompatible screen position and incompatible ball position/compatible screen position, *t*(206) < 1, *p* = .940. The lowest error rate was found in trials with compatible ball and screen positions (*M* = 0.95%; *SEM* = 0.11%) and the highest error rate was found in trials with incompatible ball and screen positions (*M* = 3.13%; *SEM* = 0.30%). The final one was an interaction of Screen Position Compatibility and Traffic Directionality, *F*(1, 203) = 7.44, *p* = .007, ƞ_p_^2^ = .035. Mirroring the RT findings, this outcome indicated that the global Simon effect in error rates differed between left-hand traffic participants (Australia, Malaysia) and right-hand traffic participants (China, Germany; *M* = 1.43%; *SEM* = 0.20% vs. *M* = 0.71%; *SEM* = 0.16%, respectively), *t*(203) = 2.86, *p* = .005.

In addition, a GLMM was run on trial-wise accuracy data with the following model specifications: glmer(Accuracy ~ Screen Position Compatibility * Ball Position Compatibility * Traffic Directionality * Cultural Orientation + (1 + Display Size | ParticipantID), family = ‘binomial’, control=glmerControl(optimizer="bobyqa")).

This model yielded the same fixed effects and interactions as the corresponding ANOVA. There was a fixed effect of Screen Position Compatibility, χ^2^(1) = 113.90, *p* < .001, showing that performance accuracy for compatible trials was higher than for incompatible ones. There was also a significant fixed effect of Ball Position Compatibility, χ^2^(1) = 127.24, *p* < .001, showing likewise better accuracy for compatible trials than for incompatible trials. There was a significant main effect of Display Size, χ^2^(1) = 20.50, *p* < .001, with higher performance accuracy for the nine-manikin display than the one-manikin display. Further, the interaction of Display Size and Screen Position Compatibility was significant, χ^2^(1) = 20.67, *p* < .001, and there was an interaction of Screen Position Compatibility and Traffic Directionality, χ^2^(1) = 22.98, *p* < .001. The GLMM showed additional effects that were not observed in the ANOVA. More specifically, there was a fixed effect of Cultural Orientation, χ^2^(1) = 4.23, *p* = .039, indicating higher accuracy for individualistic cultures (Australia, Germany). The interaction of Ball Position Compatibility and Cultural Orientation was significant, χ^2^(1) = 29.06, *p* < .001, showing higher accuracies for individualistic cultures in the local Simon effect based on Ball Position (Australia, Germany). There was also an interaction between Ball Position Compatibility, Traffic Directionality, and Cultural Orientation, χ^2^(1) = 9.06, *p* = .003, showing lowest accuracies for the collectivistic culture with right-hand traffic (China) in the local Simon effect based on Ball Position.

## Discussion

The aim of this study was to test whether cultural differences, including variations in cultural orientation and traffic directionality, influence the Simon effects indicative of differences at the level of response selection. Our study revealed both cognitive universals and diversity. In the following, we discuss both lines of findings separately.

### Cultural universals in the Simon effects

Given human cognitive universals in regard to spatial compatibility (for overview, see Proctor & Vu, 2010), we expected substantial global and local Simon effects in all participating cultures. This is exactly what we observed: concurrent and reliable Simon effects associated with global aspects of the stimulus position (i.e., the manikin/ball’s position relative to the center of the screen) and local aspects of the stimulus position (i.e., the ball’s position relative to the manikin). These findings demonstrate parallel automatic spatial coding in two different reference frames, self-centered and object-centered. In all countries, these two Simon effects occurred in RTs as well as error rates, and were found with different analytic approaches. These results illustrate that the very same reference frames were used automatically and spontaneously in all countries without any prompting of participants.

This finding is consistent with other studies in which we have explored the processes underlying these two simultaneously occurring Simon effects based on different reference frames (Baess & Bermeitinger, 2022; Baess et al., 2022). However, the finding of distinct Simon effects is not ubiquitous. For example, the possibility of distinct Simon effects based on different reference frames has been investigated in studies aligning potential reference objects next to each other along the horizontal line, but distinct Simon effects were only found with iterative stimulus presentation and informative target-location cues (see Rubichi et al., 2006). Wang et al. (2016) used an approach similar to the one used here, presenting stimuli that allowed two different reference frames, but only observed the emergence of global and local Simon effects when drawing their (Chinese) participants’ attention to the two different reference frames. Here, we consistently observed two simultaneous Simon effects in all countries without alerting participants in any way.

The cross-cultural emergence of global and local Simon effects seems particularly interesting as the use of reference frames in language varies greatly between different countries and language families (for review, see Majid et al., 2004). The languages spoken by the participants differed not only between the four countries groups but also within one group (see OSM Table 1). Thus, despite potential differences in the relevance of reference frames across the languages spoken, distinct spatial reference frames for self-centered and object-centered coding of the task-relevant feature (i.e., “the ball”) were used. This finding illustrates that spatial and linguistic reference frames are, at least partially, distinct from one another. The spatial reference frames are very stable, although specific underlying parameters may differ based on several factors, such as previous experience with travel, navigation, and wayfinding, but also culture (for an overview, see Kitchin & Blades, 2002; Mondschein et al., 2010).

Another universal finding in all groups and analyses was the influence of visual display size (i.e., one manikin vs. a perceptual group of nine manikins), with faster responses and higher accuracy rates when a perceptual set of nine manikins was displayed. This finding is somewhat surprising given the strong differences in visual scene detection between East Asians and Westerners reported in the literature (for review, see Nisbett & Masuda, 2003; Nisbett & Miyamoto, 2005). Most of the studies investigating differences in visual scene detection have emphasized cultural differences in object categorization or in the way attention is directed to salient objects or the broader scene. East Asians tend to focus more on background objects or contextual information, whereas Westerners put more emphasis on foreground or salient objects. However, the rather simple visual scene used in the present study when presenting a perceptual group of nine manikins did not lead to any RT differences between the different countries. Evidence for differences in the allocation of attention have been shown for rapidly presented targets between Westerners and East Asians (Boduroglu & Shah, 2017; Boduroglu et al., 2009). The absence of RT differences in the present study may relate to the fact that our manikin displays neither had an inherent division between focal and background objects as parts of a visual scene nor required any change detection.

Interestingly, the modulation of the global but not the local Simon effect in both RTs and error rates depending on the display size appeared in all four samples. A larger global Simon effect was observed when an individual stimulus was presented. This result replicates our ongoing work (Baess & Bermeitinger, 2022; Baess et al., 2022). These differences in the size of the global Simon effects based on the display size suggest that the underlying event files formed both for the stimulus (i.e., regarding its screen position as left or right or regarding its color as yellow or blue) and the response selection (i.e., left vs. right) apparently differed between the two sizes of display. It has been suggested that certain event files (i.e., the representational format of stimulus and response codes) can receive more weight based on some intention or goal-related dimensions (Memelink & Hommel, 2013). This suggestion might explain why the differences in the global Simon effects were obtained in regard to the different display sizes. However, this notion also suggests that the mechanisms involved in attributing different levels of salience to different event files are rather universal, as the differences in the global Simon effect were evident in all samples. This interpretation in terms of cognitive universals is corroborated further by the lack of interaction of display size with cultural orientation or traffic directionality across all analyses. This finding is not what we expected, as the display size itself should bring about differences based on cultural orientation and cognitive style (Boduroglu et al., 2009; Masuda & Nisbett, 2001; Nisbett & Miyamoto, 2005). There seem to have been no discernible cultural influences, neither of cultural orientation nor traffic directionality, on participants’ ability to perceive the visual group display holistically. At first glance, these results are at odds with previous reports of cultural differences in perception; however, on closer inspection they might simply highlight that perceptual differences reported elsewhere did not take into account later stages of information processing that involve response selection.

### Cultural diversity in the Simon effects

However, our study also found, as hypothesized, some compelling group differences in the global and local Simon effects across both visual display types, supporting the idea of human cognitive diversity and cross-cultural differences. First, the size of the global Simon effect was modulated by traffic directionality in RTs and error rates in all analyses, as hypothesized, with larger effects observed in participants from countries with left-hand traffic (i.e., Australia and Malaysia) than those from countries with right-hand traffic (i.e., China and Germany). This effect was further qualified by the importance of cultural orientation in the non-normalized RT analyses, suggesting differences in the global Simon effect between individualistic (Germany) and collectivistic (China) cultures with right-hand traffic.

These findings are particularly insightful as traffic directionality has – to the best of our knowledge – never been identified as a source of variation in tasks exploring spatial cognition, although its impact has been conclusively demonstrated in research on traffic safety (Linkov & Zámecník, 2020; Thompson & Sabik, 2018). As shown, the spatial codes representing both Simon effects are rather universal, as the underlying spatial properties are similar regardless of where a participant lives. However, the larger global Simon effects for left-hand traffic subsamples show that the response conflict elicited by SR-incompatible trials is more pronounced for left-hand traffic participants. The underlying source of this variation is unclear; we can only speculate that the observed differences might reflect differences in the underlying spatial features of the urban environment, which over the long term may modify aspects of spatial cognition (Kitchin, 2015; Kitchin & Blades, 2002). This finding also means that the effects reported by Samuel et al. (2018), who attributed differences in Simon effects between Koreans and Brits to differences between collectivistic and individualistic cultures, may also be explained by differences in traffic directionality.

Other factors that have received some attention in this domain are writing direction and the spatial representation of time. Writing direction (i.e., horizontally left-to-right, horizontally right-to-left, or vertically top-to-bottom) has been found to modulate spatial compatibility effects (Chan & Bergen, 2005; Vallesi et al., 2014) and might thus also influence the Simon effect. We could not assess this factor because participants in our study mostly used left-to-right reading and writing directions. Other studies showed an influence of the representation of time (i.e., conceptualization along a horizontal or vertical timeline) on a spatial priming task (Boroditsky, 2001). Chen et al. (2013) reported an overall vertical bias in temporal judgments in Taiwanese but a greater horizontal effect in Chinese participants, in line with predominantly vertical versus horizontal printing practices in Taiwan and mainland China, and thus differences in lifetime reading experiences. This work on differences at the interplay between time and space is important in that it shows how spatial cognition is modulated by long-term impact of different reference systems used for expressions of time (see also Gu et al., 2019). Thus, a diverse range of factors has been shown to influence performance on spatial tasks, including writing direction and time representation and even congruence of the altitudes of a person’s place of residence and the place of testing (Bondi et al., 2021). Our novel finding of a dependency of the global Simon effect on traffic directionality shows that this factor may also be an important one. The influence of traffic directionality is potentially further shaped by cultural differences along the collectivism-individualism spectrum (as shown in our data by the three-way interaction) or other cultural differences, such as writing direction or ways of thinking about time.

Furthermore, the size of the local Simon effect was seemingly influenced by cultural orientation in the analysis of non-normalized RTs and the mixed-effects modeling of RTs and error rates. Larger local Simon effects were observed for participants living in collectivistic cultures (i.e., China and Malaysia), which is in line with our hypotheses. This finding adds to the existing literature reporting differences in visual scene detection between Westerners and East Asians (for overview, see Nisbett & Masuda, 2003; Nisbett & Miyamoto, 2005). This line of research has shown – in an integrative perspective across different experimental tasks – that people in collectivistic countries detect more changes in the field and relationships between objects. Therefore, as the reference frame underlying the local Simon effect is an object-centered one, the interference from an incompatible stimulus position in regard to the response assignment (i.e., stimulus left of manikin but right responses required) might have been more pronounced in East Asians focusing on the local details of the stimuli. Although there was no interaction with display size, this interference seemed more pronounced in both East Asian countries with the larger display size (Ball Compatibility Effects: 12 ms and 13 ms for China and Malaysia vs. 7 ms and 9 ms for Australia and Germany). Thus, East Asians might implicitly process these different object-centered relations better, especially when a more complex visual display is presented.

### Limitations

Our study revealed robust Simon effects in each of the four samples as well as a modulation of the global Simon effect based on the visual display size. Further, it revealed fine-grained cultural differences in the global Simon effect based on traffic directionality and in the local Simon effect based on cultural orientation. However, there are also potentially relevant factors that we could not control for that could limit the generalizability of the present results.

A limitation of the present study is that cultural orientation was assessed in relation to the countries’ placement on the individualism-collectivism dimension (Hofstede, 2001). Country scores on the individualism-collectivism dimension (out of 100) are 90 (Australia) and 67 (Germany) for the countries with individualistic orientation, as compared to 20 (China) and 26 (Malaysia) for the countries with collectivistic orientation (for individualism score, see Hofstede, 2015). This procedure obviously ignores inter-individual variation across participants. Self-reported measures on the individualism-collectivism dimension were only available in the Australian and Chinese samples (see OSM Table 2). Although there were clear differences between these two samples on most measures in the expected direction, future studies could consider using an individualism-collectivism measure as a predictor or as the basis for group separation. In a similar vein, the classification of traffic directionality was based on the country’s traffic directionality. This procedure likewise did not take into account individual variations or experiences. As a general note, our study included only one country for each combination of cultural direction and traffic directionality. Of course, it is necessary to show that our findings generalize to other countries varying along both dimensions by conducting a similar study protocol with more or different countries representing the variations.

Similarly, our participant groups were somewhat heterogeneous, with some participants also speaking other languages than the country’s official language or having different ethnicities. For example, the Malaysian sample mainly consisted of Malaysian Chinese participants, but there were also participants with a Malay or Malaysian Indian background. We also did not directly assess participants’ nationality, how long they had been living in their home country, or whether they had resided in or traveled to other countries. Future studies could include a measure of exposure to other cultures. We also had no direct information on the dominant language spoken by our participants as we only asked generally which languages they spoke (see OSM Table 1). However, there are differences between languages in relation to how spatial relationships are expressed (Levinson, 1996). Almost all participants reported a left-to-right writing direction, but this assessment does not completely rule out that other writing directions influenced the results (e.g., Bergen & Lau, 2012; Chan & Bergen, 2005; Chen et al., 2013). Across the four samples, gender ratios were not equal. As we did not have a specific hypothesis involving gender, we did not carefully balance the samples on this variable. However, gender differences in Simon tasks have previously been reported (Mosso et al., 2020; Stoet, 2017), thus this imbalance might have impacted the results as well.

Additionally, we used a paradigm with manikins holding colored balls to measure global and local Simon effects. This paradigm has been used in different studies with German samples (Baess & Bermeitinger, 2022; Baess et al., 2022; Baess et al., 2018), which found results identical to the ones of German participants in the present study. To ensure the cultural differences in local and global Simon effects observed in the present study are reliable, other versions of the Simon paradigm, for example one with horizontally aligned reference boxes (for review, Rubichi et al., 2006), or even other paradigms investigating response selection in spatial tasks, should be employed.

### Conclusions

Despite these potential limitations, our study highlighted remarkable universals in the Simon task across the four countries. These universals in the global and local Simon effects reveal that the underlying cognitive representations of stimulus and responses features were similarly organized across samples (for the theoretical framework, see Hommel, 2019). In spite of these universals, the sizes of the global and local Simon effects were modulated by traffic directionality and cultural orientation, respectively.

Finally, the apparent influence of traffic directionality on spatial compatibility effects calls for a shift in cross-cultural research away from the often isolated consideration of the cultural orientation variable along the individualism-collectivism spectrum to other cultural factors and their interactions. For spatial cognition, traffic directionality promises to be an interesting factor for future cross-cultural studies, which has so far been mostly neglected outside of its relevance as a source for traffic accidents (Linkov & Zámecník, 2020; Mondschein et al., 2010).

## Supplementary Information

ESM 1 (DOCX 33 kb)

## Data Availability

The datasets generated and analyzed in the current study are available from the corresponding author on request.

## Footnotes

1 The actual size on the screen varied slightly across countries due to different screen sizes.

2 All participants also completed a spatial rotation test (the card rotation test of the Kit of Factor-Referenced Cognitive Tests; Ekstrom, R. B., French, J. W., Harman, H. H., & Dermen, D. (1976). *Manual for Kit of factor-references cognitive tests*. Educational Testing Service) in paper-and-pencil (Germany, Malaysia) or electronic version (Australia, China). Results are not reported here.

3 Given the known effect of handedness on the Simon effect (e.g., Rubichi & Nicoletti, 2006), the same ANOVA was calculated with right-handed participants only (*N* = 191), yielding similar results: a main effect of Display Size, *F*(1, 187) = 69.05, *p* < .001, ƞ_p_^2^ = .270, a main effect of Ball Position Compatibility, *F*(1, 187) = 80.99, *p* < .001, ƞ_p_^2^ = .302, a main effect of Screen Position Compatibility, *F*(1, 187) = 373.72, *p* < .001, ƞ_p_^2^ = .666; a main effect of Cultural Orientation, *F*(1, 187) = 26.12, *p* < .001, ƞ_p_^2^ = .123; an interaction of Screen Position Compatibility and Display Size, *F*(1, 187) = 121.99, *p* < .001, ƞ_p_^2^ = .395; an interaction of Cultural Orientation and Traffic Directionality, *F*(1, 187) = 13.38, *p* < .001, ƞ_p_^2^ = .067; an interaction of Screen Position Compatibility and Traffic Directionality, *F*(1, 187) = 6.85, *p* = .010, ƞ_p_^2^ = .035, which was further qualified by a three-way interaction of Screen Position Compatibility, Traffic Directionality, and Cultural Orientation, *F*(1, 187) = 6.94, *p* = .009, ƞ_p_^2^ = .036; and additionally, an interaction between Display Size and Ball Position Compatibility, *F*(1, 187) = 5.04, *p* = .026, ƞ_p_^2^ = .026.

4 We also calculated an ANOVA with Screen Position (left vs. right), Ball Position (left vs. right), Response (left vs. right), and Display Size including Cultural Orientation and Traffic Directionality as between-subject factors. This analysis yielded comparable results. Importantly, there was no interaction between Response and Traffic Directionality or between Screen Position and Traffic Directionality, showing the absence of a direct influence of Traffic Directionality on Screen Position per se.

5 The pattern of results was also confirmed in an ANOVA with the same factors for right-handed persons only. This ANOVA obtained a main effect of Display Size, *F*(1, 187) = 8.36, *p* = .004, ƞ_p_^2^ = .043; a main effect of Ball Position Compatibility, *F*(1, 187) = 17.90, *p* < .001, ƞ_p_^2^ = .087; a main effect of Screen Position Compatibility, *F*(1, 187) = 62.41, *p* < .001, ƞ_p_^2^ = .250, and also a main effect of Cultural Orientation, *F*(1, 187) = 4.30, *p* = .040, ƞ_p_^2^ = .022, which was not found when including left-handers. Three interactions were observed: i.e. between Screen Position Compatibility and Traffic Directionality, *F*(1, 187) = 7.00, *p* = .009, ƞ_p_^2^ = .250; between Display Size and Screen Position Compatibility, *F*(1, 187) = 31.63, *p* < .001, ƞ_p_^2^ = .145; and between Screen Position Compatibility and Ball Position Compatibility, *F*(1, 187) = 12.15, *p* = .001, ƞ_p_^2^ = .061.
