# Supplementary material for: What Simon “knows” about cultural differences: The influence of cultural orientation and traffic directionality on spatial compatibility effects
Source: Mem Cognit. 2022 Sep 30;51(3):526–42. doi: 10.3758/s13421-022-01360-9 (PMC9992257; doi:10.3758/s13421-022-01360-9)
Supplement: Supplementary file 1 — (DOCX 33 kb) [file 13421_2022_1360_MOESM1_ESM.docx]

**Supplement Table 1**

Overview over the spoken languages and reading preferred reading directions separated by country. The data was not further clustered but represents exactly the information provided by the subjects. The items were as follows: Language – please list all, occupation – if student please specify your subject. The primary reading and writing direction of single sentences was assessed in the following way: left to right; right to left; top to bottom and bottom to top.

| Country | Languages spoken (frequencies of mentioning this language in free textfield) | Occupation | Primary reading and writing direction of single sentence |
| --- | --- | --- | --- |
| Australia | 40 English  4 Chinese Mandarin  3 French  2 Malay  2 Tamil  1 Afrikaans  1 Farsi/ Persian  1 Hebrew  1 Hiligaynon  1 Indonesian  1 Irish  1 Italian  1 Shona  1 Sinhalese  1 Tagalog | 60 students  (Acting [1];  Biochemistry & Genetics [2]  Biomedical Science [5];  Chemistry [1];  Commerce [5];  Computer Science & Engineering [4]  Economics [3]  Finance [1]  History [1];  Human Biology & Anatomy [3];  Humanities in Health and Medicine [1];  Law [3],  (HR) Management [2];  Marketing [1];  Medicine [1];  Neuroscience [4];  Political Science [1];  Psychology [21]) | 60 left to right |
| China | 51 English  51 Chinese Mandarin  4 Japanese  3 Korean  1 German | 52 students  ([Applied] Psychology [36];  Preschool Education [15]) | 50 left to right  1 top to bottom |
| Germany | 46 German  43 English  17 French  13 Spanish  6 Latin  3 Italian  3 Russian  2 Polnish  2 Turkish  1 Arabic  1 Hebrew  1 Chinese | 46 students (Psychology [46]) | 45 left to right  1 right to left |
| Malaysia | 50 English  44 Malay (Bahasa Melayu)  18 Mandarin  16 Chinese  12 Cantonese  5 Japanese  3 Tamil  3 Hakka  2 Korean  2 Urdu  1 Punjabi  1 Hindi  1 Indonesian  1 Hokkien  1 Russian  1 French  1 Farsi  1 Tajik | 4 employed (IT, research assistant, security)  44 students (Accounting [1]; Arts [4]; Biomedical Science [2]; Business & Finance [6]; Computer Science [1]; English [1]; Engineering [9]; Environmental Science [2]; Pharmacy [5]; Psychology [4]; Science [5]; not specified [4];  2 without specifying occupation) | 45 left to right  5 top to bottom |

**Supplement Table 2**

Means and standard deviations (in parentheses) for the Australian and Chinese sample in the collectivism-individualism scale (ranging from 1 [strongly disagree] to 7 [strongly agree]).

|  | Australian sample (*N* = 60) | Chinese sample (*N* = 52) | *t* value (*df*) | *p* |
| --- | --- | --- | --- | --- |
| Horizontal individualism | 5.16 (1.04) | 4.48 (1.61) | 2.61 (85) | .011 |
| Vertical individualism | 4.15 (1.07) | 3.47 (1.26) | 3.12 (110) | .002 |
| Horizontal collectivism | 5.38 (1.15) | 4.82 (1.53) | 2.66 (110) | .031 |
| Vertical collectivism | 4.20 (1.02) | 4.47 (1.36) | 1.21 (110) | .228 |
|  |  |  |  |  |

Note: The Australian sample used the 32-item version (Singelis et al., 1995) and the Chinese sample used the 28-item version (Liu, 2017) of the Collectivism-Individualism scale. Both groups were compared using grouped t-tests for each dimension. In case of a violation of the equality of variances, *t*-values and *df* are reported for the inequality of variance.

**References**

1. Liu, P. (2017). An investigation of individualist and collectivist orientations of university students. Jiangxi Normal University. https://oversea.cnki.net/KCMS/detail/detail.aspx?dbcode=CMFD&dbname=CMFD201801&filename=1017089315.nh&uniplatform=OVERSEAS_EN&v=S8PQVxJuGYaa74Bcc7SkvKbQ zzdSBJ3nOSw_PNuSnzs6Pja463-L3r5yYg9h6Z0
